# HSP70 of *Leishmania amazonensis* alters resistance to different stresses and mitochondrial bioenergetics

**DOI:** 10.1590/0074-02760160087

**Published:** 2016-06-10

**Authors:** Bárbara Santoni Codonho, Solange dos Santos Costa, Eduardo de Figueiredo Peloso, Paulo Pinto Joazeiro, Fernanda Ramos Gadelha, Selma Giorgio

**Affiliations:** 1 Universidade Estadual de Campinas, Instituto de Biologia, Departamento de Biologia Animal, Campinas, SP, Brasil; 2 Universidade Estadual de Campinas, Instituto de Biologia, Departamento de Bioquímica e Biologia Tecidual, Campinas, SP, Brasil

**Keywords:** Leishmania amazonensis, HSP70, mitochondria, oxidative stress, hyperbaric oxygen

## Abstract

The 70 kDa heat shock protein (HSP70) is a molecular chaperone that assists the parasite *Leishmania* in returning to homeostasis after being subjected to different types of stress during its life cycle. In the present study, we evaluated the effects of HSP70 transfection of *L. amazonensis* promastigotes (pTEX-HSP70) in terms of morphology, resistance, infectivity and mitochondrial bioenergetics. The pTEX-HSP70 promastigotes showed no ultrastructural morphological changes compared to control parasites. Interestingly, the pTEX-HSP70 promastigotes are resistant to heat shock, H2O2-induced oxidative stress and hyperbaric environments. Regarding the bioenergetics parameters, the pTEX-HSP70 parasites had higher respiratory rates and released less H2O2 than the control parasites. Nevertheless, the infectivity capacity of the parasites did not change, as verified by the infection of murine peritoneal macrophages and human macrophages, as well as the infection of BALB/c mice. Together, these results indicate that the overexpression of HSP70 protects *L. amazonensis* from stress, but does not interfere with its infective capacity.

Leishmaniosis is a group of diseases caused by the protozoan parasite *Leishmania*, transmitted by the phlebotomine vector. *L. amazonensis* causes localised and diffuse cutaneous lesions and is of epidemiological importance in the Amazon Region (Grimaldi Jr & Tesh 1993, McGwire & Satoskar 2014). The leishmanioses are diseases that have been neglected by the pharmaceutical industry, even though no human vaccine exists; the drugs that are available have side effects and exhibit increasing signs of resistance (Oliveira et al. 2011, Savoia 2015).

During transmission from the insect vector to the mammalian host and from the promastigote to amastigote differentiation, *Leishmania* has to address several different stresses, such as variations in pH and temperature and oxidants produced by host macrophages; the molecular chaperones known as heat shock proteins (HSP) are important for intracellular survival, differentiation and virulence (Shonhai et al. 2011). The HSP70s of *Leishmania* are part of a multigene family, and the regulation of this family occurs at the post-transcriptional level (Hunter et al. 1984, Shonhai et al. 2011, Requena et al. 2015). HSP70 is cytosolic and makes up 2.1% of the total protein content of unstressed *L. major* promastigotes (Brandau et al. 1995). Heat shock has been shown to increase HSP70 abundance (Brandau et al. 1995). Toxic oxidants (Wilson et al. 1994), tumour necrosis factor (Salotra et al. 1995) and pentavalent antimony treatment (Kumar et al. 2010) have been shown to cause an increased expression of HSP70 in *L. donovani* and *L. chagasi*. Additionally, the transfection of HSP70 in *L. chagasi* led to an increase in its resistance to H_2_O_2_ (Miller et al. 2000) and protected *L. chagasi* and *L. tarentolae* promastigotes from the effect of trivalent antimony (Brochu et al. 2004). No studies have examined the potential effect of HSP70 on energy metabolism. Although the sequence and genomic organisation of the HSP70 gene (Bock & Langer 1993), the protein’s identification using immunohistochemistry (Araújo & Giorgio 2015) and mass spectrometry (Tei- xeira et al. 2015) and a protective DNA vaccine encoding antigens and HSP70 genes (Campbell et al. 2003) have been characterised in *L. amazonensis* experimental models, little is known about the biological role of HSP70 in this parasite species. Here, the mitochondrial bioenergetics of *L. amazonensis* transfected with HSP70 were determined, and the rates of infection, the resistance to stress and the changes of the parasite’s morphology were evaluated using different stress conditions.

## MATERIALS AND METHODS

*Culture of L. amazonensis* - *L. amazonensis* (MHOM/BR/73/M2269) promastigotes, both wild type (WT) and those transfected with pTEX or pTEX-HSP70, were grown in RPMI medium containing 2 mM L-glutamine, 2 g/L NaHCO_3_, 5.957 g/L HEPES, 10.000 UI Gentamicin and 10% heat inactivated foetal calf serum (FCS) (Degrossoli & Giorgio 2007) at 26ºC in the presence of 50 µg/mL of G418 (Geneticin) (Finzi et al. 2004). While in the *log* phase, the cells were harvested by centrifugation (388 x g at 4ºC) and were washed using phosphate-buffered saline (PBS) at a pH 7.4; the number of cells was determined using a Neubauer chamber (Arrais-Silva et al. 2005).

*Construction of the pTEX-HSP70 vector* - The HSP70 gene (Gen-Bank: AAA53690.1) was amplified by polymerase chain reaction (PCR) using the primers 5’-gaattcATGACGTTCGACGGCGCCAT-3’ and 5’- actagtTTAGTCGACCTCCTCGACCT-3’ as described by Montalvo et al. (2010); the gene was then cloned into the *Eco* RI / *Spe* I restriction sites of the *pGEM™ T-Easy* vector (Promega, Madison, WI, USA) and subcloned as a 1974 pb *Eco* RI / *Hind* III fragment in the trypanosomal expression vector pTEX (Kelly et al. 1992). Cells were transformed using electroporation and were selected using G418 as described (Peloso et al. 2011). Gel analyses, Ponceau staining and western blotting using a rabbit polyclonal HSP70 antibody (SPC-103C/D) (Stress Marq Bioscience Inc.) and anti-rabbit IgG horseradish peroxidase conjugate antibody (Cell Signalling) were performed as previously described (Klein et al. 1995, Moore & Viselli 2000, Peloso et al. 2011). Densitometry analyses were made using the Image J 1.48 software.

*Ultrastructural analyses* - The parasites (10^7^) were centrifuged, and the pellets were fixed using 2.5% glutaraldehyde and 0.5% tannic acid (Electron Microscope Science, Hatfield, PA) in 0.1 M sodium cacodylate buffer (Electron Microscope Science) at pH 7.4. Afterwards, the parasites were rinsed for 10 min using the same buffer, and the cells were added to coverslips pretreated with poly-l-lysine for scanning electron microscopy. Next, the cells were post-fixed in a 1% OsO_4_ (Electron Microscope Science) solution for 45 min. For transmission electron microscopy, cells were rinsed (10 min) using a glycosylated solution and post-fixed using 1% uranyl acetate in the rinsed solution. On the next day, the cells were rinsed (10 min) using water, were placed in agarose and were dehydrated using an ethanol gradient and acetone. Next, the cells were embedded in the Epon 812 resin (Electron Microscope Science). Ultrathin sections were stained using uranyl acetate and lead citrate and were observed using a LEO 906 (Leica, Wetzlar, Germany) transmission electron microscope operated at 60 kV. For scanning electron microscopy, the cells were dehydrated using an ethanol gradient, were treated at the critical point with CO_2_, were mounted on stubs, were sputtered with a thin gold layer and were observed using a JEOL 5800 LV (Leica, Wetzlar, Germany) scanning electron microscope operated at 10 kV.

*Determination of the proliferation curve* - Promastigotes (10^6^ /mL) were added to 25 cm^2^ flasks containing RPMI medium and were kept at 26ºC. On a daily basis, an aliquot was removed and the number of promastigotes was determined using a Neubauer chamber (Arrais-Silva et al. 2005). The experiments were carried out in triplicate and were repeated independently three times.

*Oxygen uptake measurements* - The O_2_ consumption was monitored using a computer interfaced Clark-type oxygen electrode and with continuous stirring at 28ºC (Hansatech^®^ Systems Inc., Norfolk, England). The cells (5 x 10^7^ /mL) were incubated in standard intracellular reaction medium (SRM) (125 mM sucrose, 65 mM KCl, 2 mM KH_2_PO_4_, 0.5 mM MgCl_2_, 10 mM HEPES pH 7.2, 1 mM EGTA and 1 mg/mL BSA) in the presence of 40 μM digitonin and 5 mM succinate. Respiratory control ratios (RCR) (state 3/state 4) were determined after the addition of 400 μM ADP (state 3) and 1 μg/mL of oligomycin (state 4) (Silva et al. 2011). The experiments were carried out in duplicate and were repeated independently four times.

*Determination of the intracellular ATP* - The experiments were performed according to the manufacturer’s instructions using the Enzylight^TM^ ATP Assay Kit (EATP-100). Briefly, cells were harvested using centri- fugation (388 × g at 4ºC) and were washed using PBS. Luminescence was obtained from the reaction of the D-luciferin, luciferase and ATP that was released after cell lysis and was monitored using a luminometer (Packard Bioscience Company®) coupled to the Instrument Control MFC Application, version 3.02. The quantified luminescence was correlated to the ATP concentration using a standard ATP curve (Peloso et al. 2011). The experiments were carried out in triplicate and were repeated independently four times.

*Determination of H*_*2*_ *O*_*2*_ *release* - Promastigotes *(*10^7^ cells/mL) were incubated in PBS/1 mM MgCl_2_ in the presence of 5 mM succinate, 40 μM digitonin, 1 U/mL horseradish peroxidase, 25 μM Amplex Red (Molecular Probes^®^) and antimycin A (AA) (1.5 μg/mL). The fluorescence was monitored at the excitation and emission wavelengths of 563 nm and 587 nm, respectively, using a Hitachi F2500 fluorescence spectrophotometer and with continuous stirring. The quantitative correlation between the fluorescence and the H_2_O_2_ released by the cells was determined as previously described (Peloso et al. 2011). The experiments were carried out in triplicate and were repeated independently three times.

*Determination of superoxide production* - O_2_^-^ production was assayed using MitoSOX [3,8-phenanthridine diamine, 5-(60-triphenylphosphoniumhexyl)-5,6-dihydro-6- phenyl, Molecular Probes^®^]. Briefly, cells (3 x 10^8^ /mL) were loaded with 5 µM MitoSOX in Krebs-Henseleit buffer (KH buffer, 15 mM NaCO_3_, 5 mM KCl, 120 mM NaCl, 0.7 mM Na_2_HPO_4_, 1.5 mM NaH_2_PO_4_) at 28ºC. After 10 min, the cells were washed and resuspended in KH buffer. The detection of oxidised MitoSOX (oxMitoSOX) in 10^8^ cells/mL was performed in this buffer in the presence of 40 µM digitonin and 5 mM succinate. The fluorescence was detected using a Hitachi F2500 fluorescence spectrophotometer with excitation and emission wavelengths of 510 and 580 nm, respectively (Piacenza et al. 2007, Peloso et al. 2012). The experiments were carried out in triplicate and were repeated independently three times.

*Determination of the parasite resistance to stresses* - Promastigotes (10^6^ /mL) were incubated in PBS or RPMI medium (only for the heat shock treatment) and were submitted to each of the following types of stress separately. H_2_O_2_: the parasites were treated using H_2_O_2_ (0-600 µM) for 30 min. Heat shock: the parasites were incubated in RPMI at 37ºC (otherwise, they were maintained at 26ºC) for 2 h. Hyperbaric oxygen (HBO): the parasites were exposed for 2 h to 100% O_2_ at 2.5 atmospheres absolute (ATA) in a hyperbaric chamber (Research Chamber, model HB 1300B, Sechrist Inc., Anaheim, USA) (otherwise, they were maintained under normoxic conditions of 21% O_2_ at 1 ATA). After the treatments, the parasites were centrifuged and washed using PBS, resuspended in RPMI medium (10^6^ cells/mL) and maintained at 26ºC. On the third day of culture, an aliquot was removed to determine the number of parasites (Wilson et al. 1994, Finzi et al. 2004, Arrais-Silva et al. 2005). The experiments were carried out in triplicate and were repeated independently three times.

*Macrophage infection with L. amazonensis* - Primary mouse macrophages were obtained from normal BALB/c mice by peritoneal lavage, were maintained in 24-well cell cultures plates containing glass coverslips and RPMI medium supplemented with antibiotics and 10% FCS at 37ºC in a humidified incubator with 21% O_2_ and 5% CO_2_ balanced N_2_, and were infected with a ten-fold excess of promastigotes for 2 h, as previously described (Arrais-Silva et al. 2005). After the infection, the cell cultures were washed to remove extracellular parasites, and fresh medium was added. Peripheral blood mononuclear cells (PBMC) isolated from the heparinised blood of healthy human adults by centrifugation using a Ficoll Hypaque 1.077 gradient (Sigma) were cultured for seven days with RPMI medium supplemented with 25 µg/mL gentamicin and 10% FCS to achieve differentiation into macrophages; then, these cells were infected with a ten-fold excess of promastigotes for 24 h at 34ºC. After the infection, the cell cultures were incubated for an additional 24 h at 37ºC. The promastigotes used for infection were WT or those transfected with either pTEX or pTEX-HSP70; another treatment group given pentavalent antimony (16 µg/mL: *N*-methyl glucamine antimonate) (Sanofi Aventis Pharma, São Paulo, Brazil) for 24 h. For the evaluation of the percentage of infected macrophages and the number of intracellular amastigotes, approximately 600 macrophages were counted on triplicate coverslips stained with Giemsa and were examined microscopically at 1,000 magnification (Arrais-Silva et al. 2005). The experiments were carried out in triplicate and were repeated independently three times.

*Animal infection* - Female BALB/c mice (six weeks old) were obtained from the Centro de Bioterismo - Unicamp, Campinas, SP, Brazil, and 10^7^ promastigotes (WT and transfected with pTEX or pTEX-HSP70) were injected subcutaneously into the hind footpad. The lesion size was measured weekly using a calliper and was compared to the contralateral uninfected footpad (Araújo et al. 2012). The experimental protocols were approved by the Institute of Biology/Unicamp Ethical Committee for Animal Research (number 3056-1). Two independent experiments were completed using three mice per group.

*Statistical analysis* - The data are represented as the mean ± standard error and the differences between means were determined using Student’s *t*-test in Origin 6.0 software. A p value < 0.05 was considered statistically significant.

## RESULTS

**Fig. 1:**
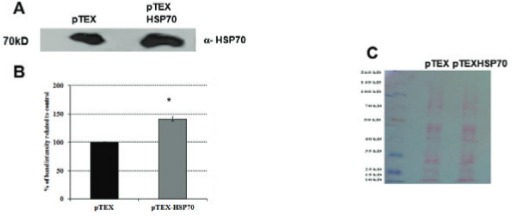
: western blotting analysis of the pTEX and pTEX-heat shock proteins (HSP)70 *Leishmania amazonensis* promastigotes. A: western blotting analysis using anti-HSP70 in *L. amazonensis* transfected with the pTEX or pTEX-HSP70 vector. Lysates from the pTEX and pTEX-HSP70 parasites were prepared and resolved using SDS-PAGE (20 µg protein/lane); B: densitometry analysis of A; C: ponceau-S staining (loading control). The best representatives of three independent experiments are shown.

**Fig. 2:**
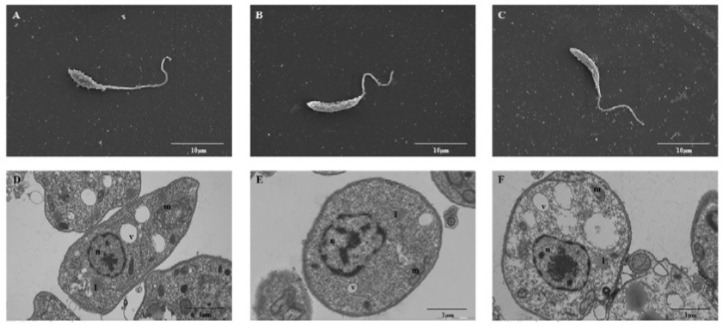
: ultrastructural analysis of the wild type (WT) and pTEX- or pTEX-heat shock proteins (HSP)70-transfected *Leishmania amazonensis* promastigotes. Electron microscopy scanning of the A: WT; B: pTEX; and C: pTEX-HSP70 parasites. Ultrathin sections of the D: WT; E: pTEX; and F: pTEX-HSP70 parasites. Abbreviations: l - lipid bodies; m - mitochondria; n - nucleus; v - vacuole. Scale bar 10 µM (A, B and C). Scale bar 1 µM (D, E and F).

Using electroporation, *L. amazonensis* were transfected with the empty vector pTEX or the cloned vector pTEX-HSP70 (pTEX and pTEX-HSP70 parasites, respectively) generating stable transformants that were stable for six months; during this time, the parasites were passaged using axenic medium and macrophages cultures. Fig. 1 shows the successful cloning of the HSP70 gene into the pTEX vector, gel electrophoresis and western blotting analysis. The pTEX-HSP70 promastigotes expressed HSP70 protein in higher levels than the pTEX promastigotes. The ultrastructural analysis of the transfected parasites was performed, and the results were compared to the parasites (WT). Scanning and transmission electron microscopy revealed that all of the typical ultrastructural characteristics of these parasites were maintained, and the organelles were well-preserved (Fig. 2).

**Fig. 3:**
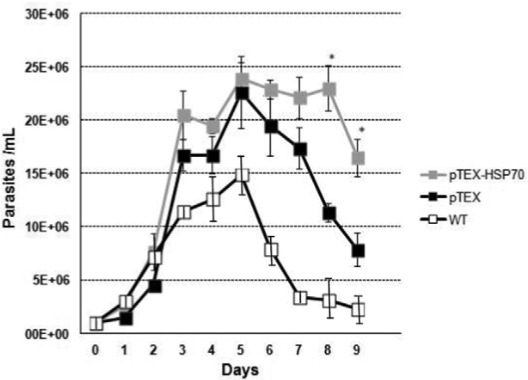
: growth curve of *Leishmania amazonensis* promastigotes. 106 cell/mL WT, pTEX and pTEX-heat shock proteins HSP70 promastigotes were incubated in RPMI medium at 26ºC. The results were obtained by the daily counting of promastigotes. Statistical analysis: *t*-test: *p < 0.05.

Fig. 3 represents the proliferation curves of the transfected and WT parasites. The pTEX-HSP70 and pTEX promastigotes showed faster and higher proliferative rates than the WT promastigotes. Notably, pTEX-HSP70 parasites remained in the stationary phase longer (four days) than the pTEX and WT parasites (three days) (Fig. 3).

Table demonstrates that the pTEX-HSP70 promastigotes were more resistant to H_2_O_2_ treatment than the pTEX promastigotes. Similar results were found with heat shock (37ºC) treatment and after hyperbaric oxygen exposure, which increased the generation of intracellular reactive oxygen species (Thom 2009).

**Fig. 4:**
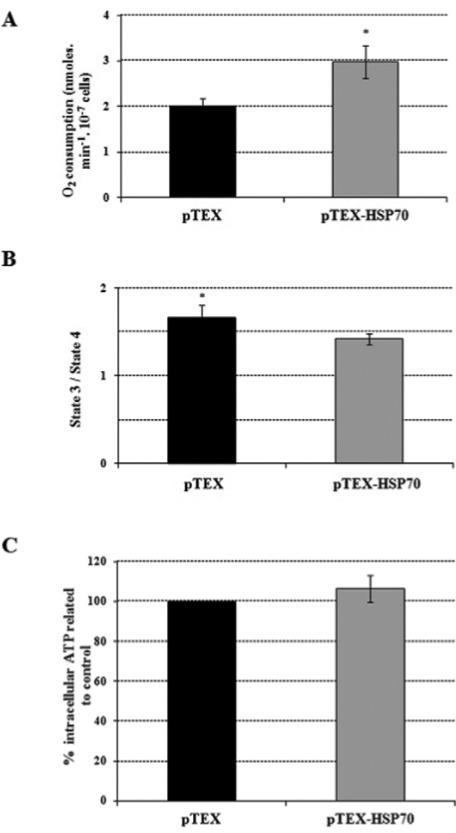
: oxygen consumption rates, respiratory control and intracellular ATP levels of the pTEX and pTEX-heat shock proteins 70 parasites. A: promastigotes were incubated in respiratory buffer, in the presence of 40 μM digitonin and 5 mM succinate; B: 400 μM ADP (state 3) and 1 μg/mL oligomycin (state 4) used for the determination of the respiratory control ratios; C: ATP levels were determined in 108 cells/mL using the manufacturer’s protocol. Statistical analysis: *t*-test: *p < 0.05.

Bioenergetic parameters were also monitored; succinate-supported oxygen consumption rates in the pTEX-HSP70 parasites were 50% higher than in the pTEX parasites (Fig. 4A). Concurrently, the RCR was determined to evaluate the level of coupling between the mitochondrial respiratory chain and oxidative phosphorylation. The oxygen consumption rates (nmoles/min^-1^.10^-7^ cells) for the pTEX promastigotes in the presence of ADP (state 3) were 2.68 ± 0.16 and in the presence of oligomycin (state 4) were 1.62 ± 0.21; in contrast, the pTEX-HSP70 promastigote values in the presence of ADP were 3.29 ± 0.32 and in the presence of oligomycin were 2.32 ± 0.26. As shown in Fig. 4B, the pTEX-HSP70 parasites had an RCR that was 15% lower compared to the pTEX parasites. As respiratory control was lower in the pTEX-HSP70 parasites, the ATP production was measured because it may have been inhibited by the observed uncoupling. The results showed that the pTEX-HSP70 parasites had an ATP production only 6% higher than that observed in the pTEX promastigotes (Fig. 4C).

**TABLE:** Ability of transfected *Leishmania amazonensis* to resist distinct types of stress

Treatments	IC_50_ H_2_O_2_ (μM)^1^	Heat shock^2^ (# cells x 10^6^/mL)		HBO^3^ (# cells x 10^6^/mL)	
		26ºC	37ºC	21% O_2_	100% O_2_
pTEX	269.5 ± 12.7	10.5 ± 0.12	6.01 ± 0.33	11.0 ± 0.49	8.1 ± 0.07
pTEX-HSP70	401.0 ± 4.9*	9.86 ± 0.26	9.74 ± 0.26*	13.0 ± 0.8	12.0 ± 0.57*

Parasites (10^6^ cells/mL) were incubated in PBS or RPMI medium (only for the heat shock treatment) and treated with H_2_O_2_ (0-600 µM) for 30 min^1^, treated for 2 h with heat shock (37ºC) or maintained at 26ºC^2^, exposed for 2 h to HBO (100% O_2_ at 2.5 ATA) or maintained under normoxic conditions (21% O_2_ at 1ATA)^3^. After the treatments, the parasites were centrifuged and resuspended in RPMI medium; on the third day of culture, the number of parasites was determined using a Neubauer chamber. The results are represented as the mean ± standard errors. Statistical analysis: *t*-test: *p < 0.05 compared to the pTEX promastigotes subjected to the same treatment.

**Fig. 5:**
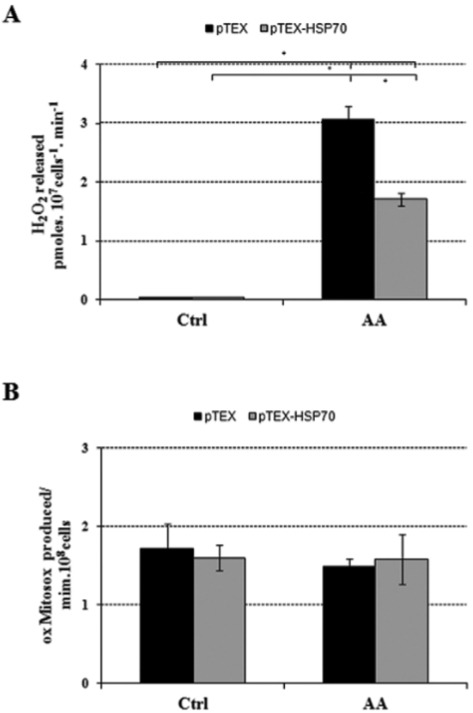
: mitochondrial H2O2 release and the production of O2- in transfected promastigotes. A: the pTEX or pTEX-heat shock proteins (HSP)70 promastigotes (107 cells/mL) were incubated in PBS/MgCl2 in the presence of 40 μM digitonin, 5 mM succinate, 1 U/mL HRP, 25 μM Amplex Red (Ctrl) and 1.5 μg/mL antimycin A (AA). B: pTEX or pTEX-HSP70 (108 cells/mL) previously loaded with MitoSox were incubated in KH buffer in the presence of 40 μM digitonin, 5 mM succinate (Ctrl) and 1.5 μg/mL antimycin A (AA).

Next, we also evaluated the H_2_O_2_ and superoxide production of these promastigotes. Under the control conditions, the pTEX and pTEX-HSP70 parasites had low rates of H_2_O_2_ release with no statistically significant differences between them (Fig. 5A). However, when an inhibitor of complex III of the mitochondrial respiratory chain, antimycin A (AA), was added to the experiment, the pTEX parasites released 80% more H_2_O_2_ than the pTEX-HSP70 parasites (Fig. 5A). Concurrently, the generation of superoxide anion (O_2_^-^) was also assessed. In contrast to H_2_O_2_ release, similar rates were observed under both control and AA-treatment conditions (Fig. 5B).

**Fig. 6:**
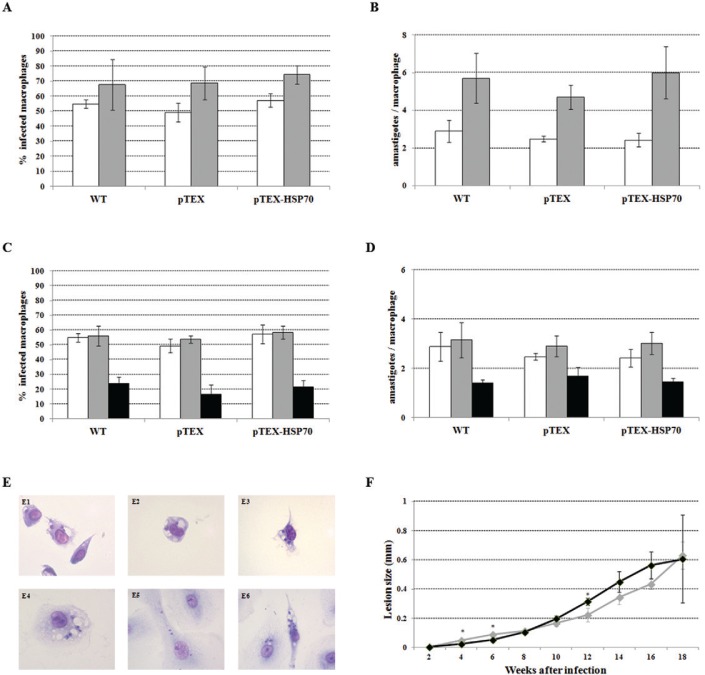
: *Leishmania amazonensis* wild type (WT), pTEX and pTEX-heat shock proteins (HSP)70 promastigotes in vitro and in vivo infectivity. A: the percentage of infected cells; and B: the number of amastigotes per macrophage were counted at 24 h in murine macrophage cultures (white) and 48 h in human macrophage cultures (grey) infected with the WT, pTEX or pTEX-HSP70 promastigotes; C: the percentage of infected cells and D: the number of amastigotes per macrophage at 24 h after infection of the murine macrophages cultures with the WT, pTEX and pTEX-HSP70 promastigotes heat shocked (37ºC) for 2 h (grey) or the WT, pTEX and pTEX-HSP70 promastigotes non-heat-shocked (white); some macrophage cultures were infected with the non-heat-shocked WT, pTEX and pTEX-HSP70 promastigotes and treated with 16 µg/mL pentavalent antimony (black); E: light microscopy images of *L. amazonensis* infected macrophages (1000x); murine macrophages infected with E1: WT; E2: pTEX or E3: pTEX-HSP70 promastigotes; human macrophages infected with E4: WT; E5: pTEX or E6: pTEX-HSP70 promastigotes; F: lesion size of the BALB/c mice infected with 107 of the pTEX (black) and pTEX-HSP70 (grey) promastigotes in the right footpad was measured weekly and expressed as the difference in size between the infected and contralateral uninfected footpads. Statistical analysis: *t*-test: *p < 0.05.

Macrophages from two different sources, primary cells from the peritoneum of mice and human macrophages derived from peripheral blood, were used in the experiments measuring the infectivity of the promastigotes. Fig. 6A-B shows that the WT, pTEX and pTEX-HSP70 promastigotes similarly infected murine and human macrophages. The percentage of the infected macrophages and the number of the intracellular amastigotes were similar between the macrophage cultures infected with the WT, pTEX and pTEX-HSP70 promastigotes. Morphological changes expected in *Leishmania* infected macrophages (vacuoles containing amastigotes, large cell size and pseudopods) were observed in the infected cells of all groups (Fig. 6E). Therefore, these results indicate that the transfection with the vector pTEX-HSP70 does not interfere with the in vitro infectious abilities of these parasites.

Because the pTEX-HSP70 promastigotes were more resistant to heat shock (Table), we also examined whether this type of stress affected the infective ability of the parasites. Fig. 6C-D shows that the WT, pTEX and pTEX-HSP70 promastigotes previously treated using heat shock infected murine macrophage cultures at similar rates.

Next, we tested the in vivo infectivity of the pTEX and pTEX-HSP70 parasites in BALB/c mice, an inbred mouse strain susceptible to *L. amazonensis*. Fig. 6F shows that the lesions progressively increased in size in the mice inoculated with both promastigotes. Although no differences in the parasite loads obtained from the lesions were observed (2.43 x 10^9^ ± 0.28 amastigotes/g lesion tissue and 2.85 x 10^9^ ± 0.16 amastigotes/g lesion tissue for the animals infected with the pTEX and pTEX-HSP70 parasites, respectively), there were points in the course of the disease in which significant differences were observed; in mice inoculated with the pTEX-HSP70 promastigotes, the lesions were larger for the first six weeks than those inoculated with the pTEX promastigotes, and the lesions differed in size at the 12th week. From then until the observations were discontinued on the 18th week, no differences were found in the lesion sizes among the animals.

Data in the literature show that the HSP70 protein of *L. braziliensis*, *L. panamensis, L. donovani, L. tarentolae* and *L. infantum* is associated with resistance to pentavalent antimony (Brochu et al. 2004, Biyani et al. 2011, Peláez et al. 2012, Matrangolo et al. 2013). Thus, macrophages were infected with the WT, pTEX or pTEX-HSP70 promastigotes and were treated with pentavalent antimony to test their resistance to the drug. As expected, pentavalent antimony was toxic to the intracellular WT parasites, i.e., it decreased the percentage of the infected macrophages and the number of intracellular parasites in macrophage cultures infected with the WT *L. amazonensis* (Fig. 6C-D). The effects of pentavalent antimony on the pTEX- and pTEX-HSP70-infected macrophages were similar to those observed in the WT-infected macrophages (Fig. 6C-D).

## DISCUSSION

To date, studies have focused on the *L. amazonensis* sequence and genomic organisation of HSP70, as well as its identification (Teixeira et al. 2015) and detection (Araújo & Giorgio 2015). The biological role of HSP70 in this parasite species and its involvement in energy metabolism has not yet been investigated. To address this issue, we conducted the current investigation using promastigotes transfected with HSP70. The shuttle vector used in this study was developed specifically for trypanosomes and *Leishmania* (Kelly et al. 1992), and the transformed cells were stable for six months during passage using axenic medium and macrophage cultures. As noted by Kelly et al. (1992), the transformed phenotype obtained with this procedure leads to the generation of stable transformants for at least six months in the absence a drug and can be maintained through *T. cruzi* life’s cycle. The abundance of HSP70 in the pTEX-HSP70 promastigotes was similar to that obtained for *L. chagasi* HSP70 transfected with HSP70 (Miller et al. 2000). Ultrastructural examination has revealed no apparent abnormalities in the pTEX-HSP70 promastigotes. Although Folgueira et al. (2008) observed aberrant forms in HSP70 deficient/mutant *L. infantum* cultures, suggesting that the loss of a single allele of HSP70 resulted in unusual morphology, none of the previous studies of HSP70 transfected *Leishmania* (*L. chagasi* and *L. tarentolae*) (Miller et al. 2000, Brochu et al. 2004) mentioned morphological or ultrastructural abnormalities.

Interestingly the pTEX-HSP70 promastigotes remained in the stationary phase longer than the control promastigotes (pTEX and WT). Studies using an HSP70-expressing leukaemia T-cell line revealed increased viability, and in MCF-7 breast cancer cells, HSP70 transfection altered the growth curve and increased proliferation (Mosser et al. 1997, Barnes et al. 2001). None of the previous studies of *Leishmania* (*L. chagasi* and *L. tarentolae*) (Miller et al. 2000, Brochu et al. 2004), mentioned differences in the growth of the parasites. However, in these studies, G418 was not used to select the transformants, an obligatory condition when pTEX is used as a vector. Interestingly and supporting our results, the same effect on growth has been observed with other kinetoplastids, *T. cruzi* and *L. donovani* (Zhang & Matlashewski 1997, Finzi et al. 2004), suggesting that a more resistant population was selected. On the other hand, repeated failures in obtaining a null mutant line by gene replacement point to an essential role of the chaperones for survival and proliferation (Requena et al. 2015); in fact, a decreased number of parasites was detected in HSP70 deficient/mutant *L. infantum* promastigote cultures in the stationary phase (Folgueira et al. 2008). Thus, we can hypothesise that HSP70 transfection assists *L. amazonensis* promastigotes to live longer when nutrients become depleted (stationary phase), i.e., when subjected to nutritional stress.

In fact, the pTEX-HSP70 parasites exhibited the increased resistance to different stress conditions such as heat shock (37ºC), HBO exposure, which increases intracellular generation of reactive oxygen species (Thom 2009), and H_2_O_2_ treatment. In support of these data, the transfection of HSP70 in *L. chagasi* led to an increase in the resistance to H_2_O_2_ (Miller et al. 2000). There is evidence indicating that heat shock is associated with an increased HSP70 expression in several species of *Leishmania* (Wilson et al. 1994, Streit et al. 1996). In a mouse neuroblastoma cell line, it was shown that HBO promotes the increased expression of HSP70 (Shyu et al. 2004). The mechanisms by which HSP70 protects the cells from an oxidative environment and thermal stress effects are not completely understood. However, it is known that HSP70 can protect cells from lipid peroxidation, a process initiated by ROS (Shyu et al. 2004), is involved in the suppression of apoptosis through caspases expression (Ueng et al. 2013) and assists in the refolding of misfolded proteins generated in stressful environments (Sharma et al. 2010); together, these events confer a greater resistance to a variety of stresses on the pTEX-HSP70 parasites.

No studies have examined the potential effect of HSP70 on *Leishmania* energy metabolism. The bioenergetics parameter studies revealed that the pTEX-HSP70 promastigotes had increased oxygen consumption and had a slight mitochondrial uncoupling without affecting the production of intracellular ATP. There are reports that the overexpression of the HSP70 protein in HeLa cells affects their energy metabolism by decreasing the activity of the enzymes involved in oxidative phosphorylation without affecting the production of intracellular ATP (Wang et al. 2012); it is possible that similar events could be occurring in the pTEX-HSP70 promastigotes. Additionally, the electron transport chain is a major source of cellular ROS, and one of the ways cells manage ROS levels is through uncoupling mechanisms that reduce the production of ROS without affecting electron transfer (Kowaltowski et al. 2009). In this sense, while the mechanism remain unknown, HSP70 could lead to an increase in the O_2_ consumption rates, which will result in less ROS being produced. These events could be one possible tactic that parasites that overexpress proteins could use to combat the production of intracellular ROS by mitochondrial uncoupling. To test this hypothesis, we evaluated H_2_O_2_ and superoxide production. Under control conditions the pTEX and pTEX-HSP70 promastigotes had low rates of H_2_O_2_ release but when antimycin A, an inhibitor of complex III of the respiratory chain, was added to the incubation medium, the pTEX-HSP70 parasites released higher H_2_O_2_ concentrations without affecting the generation of superoxide anion. Supporting these results, previous studies have shown that HSP70 modulates the activity of glutathione peroxidase, an antioxidant enzyme, in the MDCK cell line (Guo et al. 2007); glutathione peroxidase is an anti-oxidant enzyme present in the mitochondria of *Leishmania*, which could be responsible for lowering the levels of released H_2_O_2_.

Because the pTEX-HSP70 promastigotes are more resistant to the oxidative stress generated by H_2_O_2_, we hypothesised that these parasites could be more resistant to the host cell oxidative environment. Assays of infection with the WT, pTEX and pTEX-HSP70 promastigotes in in vitro models indicate that HSP70 transfection does not interfere with or increase the capacity of transfected parasites to enter and survive within macrophages. Likewise, HSP70 transfection does not alter their infectivity in the in vivo model of BALB/c mice, an inbred mouse strain susceptible to *L. amazonensis* (Giorgio et al. 1998), although the lesions of mice infected with the pTEX-HSP70 parasites were larger for the first six weeks after inoculation. There are no reports in the literature analysing the infective rates of *Leishmania* transfected with HSP70 (Miller et al. 2000, Brochu et al. 2004), but the infectivity of HSP70 deficient/mutant *L. infantum* was reduced in macrophages and in BALB/c mice (Folgueira et al. 2008); these observations were attributed to a defect in growth detected in the mutant amastigotes and promastigotes (Folgueira et al. 2008).

Because the pTEX-HSP70 promastigotes did not show altered infectivity but were more resistant to heat shock, we examined whether this type of stress affected the infectivity of *L. amazonensis*. The observation that the induction of heat shock stress in the WT, pTEX or pTEX-HSP70 promastigotes before infection did not affect the ability of the parasites to enter and survive within macrophages indicated that, although HSP70 overexpression in stressful conditions is important, as demonstrated by our experiments of resistance in promastigotes subjected to different stresses (H_2_O_2_, heat shock and HBO), it should not be considered as the exclusive mechanism used by parasites to more efficiently return to homeostasis during infection.

In this study we did not observe the resistance of the HSP70 transfected promastigotes to pentavalent antimony; in fact, the drug was equally toxic to the WT, pTEX or pTEX-HSP70 parasites inside macrophages. Data in the literature shows that *L. tarentolae* and *L. infantum* transfected with the HSP70 gene were more resistant to antimony (Brochu et al. 2004); one reason for the different results obtained with *L. amazonensis* might be that our experiments were performed using amastigotes within macrophages, and the cited study used the axenic promastigote form.

In summary, our results indicate that HSP70 transfection in *L. amazonensis* alters the mitochondrial bioenergetics and protects against oxidative and thermal stresses encountered by the parasite during their life cycle. Future studies examining the interaction of *Leishmania* HSP70 with other chaperones families and host proteins would be useful for understanding how this parasite maintains homeostasis, which could be useful while developing suitable drug treatments.
